# Neural Correlates of Processing Negative and Sexually Arousing Pictures

**DOI:** 10.1371/journal.pone.0045522

**Published:** 2012-09-20

**Authors:** Kira Bailey, Robert West, Kellie M. Mullaney

**Affiliations:** 1 Department of Psychological Sciences, University of Missouri – Columbia, Missouri, United States of America; 2 Department of Psychology, Iowa State University, Ames, Iowa, United States of America; CSIC-Univ Miguel Hernandez, Spain

## Abstract

Recent work has questioned whether the negativity bias is a distinct component of affective picture processing. The current study was designed to determine whether there are different neural correlates of processing positive and negative pictures using event-related brain potentials. The early posterior negativity and late positive potential were greatest in amplitude for erotic pictures. Partial Least Squares analysis revealed one latent variable that distinguished erotic pictures from neutral and positive pictures and another that differentiated negative pictures from neutral and positive pictures. The effects of orienting task on the neural correlates of processing negative and erotic pictures indicate that affective picture processing is sensitive to both stimulus-driven, and attentional or decision processes. The current data, together with other recent findings from our laboratory, lead to the suggestion that there are distinct neural correlates of processing negative and positive stimuli during affective picture processing.

## Introduction

Event-related brain potentials (ERPs) have been widely used to examine the time course of neural activity associated with affective picture processing [1], [2]. (For a review see [3]). ERPs provide an index of neural activity with subsecond temporal resolution that is associated with information processing related to perception, attention, memory and emotion [4]. The literature consistently reveals differential neural activity related to the affective valence of pictures beginning as early as 100 ms after stimulus onset and persisting for the next 1000 to 2000 milliseconds (ms) [5], [6]. This research reveals that various components of the ERPs (e.g., N1, early posterior negativity (EPN), late positive potential (LPP)) may be differentially sensitive to the processing of positive and negative valence related to emotion [3], [7]–[9]. The current study expands upon recent research (e.g. [1], [2], [10]) and was designed to determine whether or not positive and negative valence are associated with different patterns of neural recruitment during affective picture processing.

### ERP Correlates of Affective Picture Processing

Studies examining the influence of emotional valence on early components of the ERPs related to visual processing (e.g., N1 and P2) have yielded mixed results [3]. Some studies reveal that the N1 is sensitive to valence [1], [5], [11], while other studies have failed to reveal an effect of valence on the amplitude of the N1 [6]. The amplitude of the posterior P2 is also greater for positive and negative pictures than for neutral pictures in some studies [6], [7].

Following the N1 and P2, the EPN reflects a transient negativity over the posterior region of the scalp between 200 and 300 ms after stimulus onset. The EPN distinguishes valenced pictures from neutral pictures and is sensitive to the level of arousal associated with the stimulus [5], [12]. Specifically, the amplitude of the EPN is greater for highly arousing pictures than for less arousing pictures that have the same valence [12]. The EPN is thought to be associated with processes related to the allocation of attention to affectively arousing stimuli that facilitates further processing [13].

The LPP represents a sustained potential that extends from the central to the parietal region of the scalp that tends to differentiate negative and positive pictures from neutral pictures [5], [14]–[16]. The LPP typically begins around 300 ms after stimulus onset and persists for the next 1000 to 2000 ms. A recent study using temporal-spatial principal components analysis (PCA) demonstrated that the LPP is comprised of three distinct components of the ERPs whose distributions extend from the central to occipital regions of the scalp [8]. One of these reflects the P3, and the other two reflect components of the ERPs that are distributed over the central and occipital regions of the scalp and are sensitive to valence. The effect of valence on the LPP interacts with the relevance of affective information to task performance, being greater when emotion is relevant [17]. This finding indicates that both bottom-up stimulus driven processes and top-down attentional or decision processes likely contribute to the generation of the LPP.

The effect of valence on components of the ERPs is thought to result from the allocation of attention to stimuli that have motivational or evolutionary significance [13]. Consistent with this idea, Schupp et al. [18] found that the amplitude of the LPP elicited by positive and negative pictures increased with the level of arousal ascribed to the pictures. The effect of valence has been observed regardless of whether or not the emotional content of the pictures is relevant to task performance [5], [6] or processing resources are consumed by a secondary task [15]. These findings led to the suggestion that automatic or bottom-up influences contribute to the effects of valence on affective picture processing. In addition to these findings, other work reveals that the amplitude of the LPP is greater when attention is directed toward arousing areas of negative pictures relative to when attention is directed toward non-arousing areas of these same stimuli [17], [19], [20]. This finding leads to the suggestion that the effects of valence on components of the ERPs are also sensitive to top-down attentional or decision processes related to affective information processing [20].

### ERPs and the Negativity Bias

In an early study using ERPs to examine the neural correlates of affective picture processing, the P3 or LPP was found to increase in amplitude from neutral pictures to positive pictures to negative pictures [21]. This finding has been replicated in more recent work [9], [22], and has been offered as physiological evidence for a negativity bias associated with affective and social information processing [21], [23]. The negativity bias is thought to result from the activation of an automatic vigilance mechanism when negative stimuli are encountered that serves to enhance the processing of information that is relevant to survival [21], [24], [25]. In contrast to the findings of these studies, some investigators have failed to observe a negativity bias in the LPP (i.e., the amplitude of the LPP was similar for positive and negative pictures; [16], [18]); and others have reported a positivity bias, wherein the amplitude of the LPP was greater for positive pictures than for negative pictures [7]. These findings may be taken to indicate that the negativity bias does not reflect a distinct component of affective information processing.

The findings of two recent studies led to the suggestion that the negativity bias observed for the LPP during affective picture processing may represent a stimulus artifact [1], [2]. Briggs and Martin [2] reported that the amplitude of the LPP was greater for unpleasant (i.e., negative) pictures than for pleasant (i.e., positive) pictures that did not include erotic images, consistent with a negativity bias. However, the amplitude of the LPP was greater for erotic pictures than for negative pictures. Based upon this finding, the authors concluded that the presence or absence of a negativity bias in previous studies was driven by whether or not erotic pictures were included in the set of positive pictures. Weinberg and Hajcak [1] examined the effect of picture content including a range of positive and negative stimuli on the amplitude of the LPP as well as earlier components of the ERPs. In an overall comparison of positive, negative, and neutral pictures these authors observed a negativity bias in the LPP, consistent with previous research [9], [21]. However, the comparison of specific categories of positive and negative pictures revealed that the amplitude of the LPP was similar for erotic pictures and pictures of mutilations, and that this was also the case when positive affiliative pictures were compared to threatening pictures. Additionally, the amplitude of the LPP for exciting/sports pictures, which are positive in valence, was significantly reduced relative to other types of positive pictures. Based upon these findings, Weinberg and Hajcak argued that the negativity bias observed in earlier studies likely resulted from the inclusion of exciting/sports pictures and the exclusion of erotic pictures in the positive picture set.

In a study examining the association between video game experience and affective picture processing, Bailey et al. [10] found that there were differences in the neural correlates of processing negative and positive pictures that reflected both early transient (e.g., EPN) and later sustained (e.g., LPP) components of the ERPs. In this study there were differences in the topography of later sustained activity depending upon picture valence, with the amplitude of the LPP being greater for negative pictures over the parietal-occipital region and the greater for positive pictures over the central-parietal region. Additionally, the neural correlates of processing negative pictures were sensitive to both the relevance of emotion to task performance and individual differences in video game experience [10]. Complimenting these findings, other recent evidence demonstrates that the amplitude of LPP elicited by aversive pictures is reduced with the addition of a working memory load to the task and that this effect interacts with individual differences in state anxiety [26]. Together, these data demonstrate that the neural correlates of processing negative pictures, and possibly the negativity bias, are sensitive to top down attentional or decisional influences that vary with task demands and personal experience rather than being strongly automatic.

### The Current Study

The current study was designed to determine whether or not there are components of the ERPs that distinguish positive and negative pictures during affective picture processing. In the study, individuals viewed positive, negative, and neutral pictures representing different categories from the International Affective Picture System (IAPS; [27]) and rated the pictures on one of three dimensions (how colorful, pleasant, or threatening the picture was). The three rating tasks were included to determine whether variation in the relevance of emotion to task performance influenced the neural correlates of processing negative and positive pictures, and allowed us to determine whether differences in neural recruitment between negative and positive pictures were sensitive to automatic and attentional or decisional influences [6], [15], [19], [20].

In addition to examining the effect of picture content and orienting task on the N1, P2, EPN and LPP with conventional univariate analyses (i.e., ANOVA), Partial Least Squares analysis (PLS; [28], [29]) was used to examine differences in the spatiotemporal distribution of components of the ERPs that were sensitive to picture content. PLS analysis is a multivariate statistical technique, similar to PCA [30], that allows one to decompose the full time course and topography of the scalp recorded ERPs into a set of orthogonal latent variables that capture differences in mean amplitude between task conditions across time and space. The use for this type of analysis is motivated by work demonstrating that the LPP may reflect the activity of multiple ERP components that could be differentially sensitive to aspects of positive and negative valence related to emotion [8], [10].

## Methods

### Ethics Statement

This study was approved by the Office of Responsible Research at Iowa State University.

### Participants

Forty undergraduates (20 females) from Iowa State University participated in this study. The participants were on average (*M* = 20.45, *SD* = 2.55) years of age. There were 34 right handed, four ambidextrous, and two left handed individuals in the sample. The results did not change when left-handed participants were excluded, so the reported results include data from all participants.

### Materials

The stimuli for the picture-rating task were selected from the IAPS [27]. Pictures were chosen based on the following criteria. Valence ratings from the normative data set were used to divide the pictures into those eliciting negative, neutral, and positive affect. The set was further divided so that negative and positive pictures had similar arousal ratings (Table 1). The remaining pictures were then coded for content. A total of 180 pictures (20 in each category) were selected that included nine types of pictures [IAPS numbers for the stimuli in the picture rating tasks: Neutral pictures (objects: 7006, 7009, 7010, 7020, 7025, 7034, 7040, 7046, 7050, 7055, 7059, 7150, 7175, 7211, 7224, 7234, 7235, 7700, 7705, 7950; animals: 1022, 1026, 1030, 1080, 1101, 1114, 1121, 1230, 1230, 1240, 1313, 1321, 1390, 1560, 1670, 1675, 1931, 1935, 1945, 1947; people: 2191, 2215, 2235, 2305, 2410, 2485, 2487, 2579, 2593, 2595, 2597, 2635, 2830, 2870, 4605, 7493, 7496, 7506, 8311, 8465). Positive pictures (erotic: 4607, 4608, 4611, 4643, 4651, 4652, 4653, 4656, 4664, 4666, 4672, 4676, 4677, 4680, 4681, 4683, 4690, 4694, 4670, 4810; positive: 2058, 2071, 2154, 2160, 2209, 2216, 2311, 2340, 2345, 2346, 4599, 4626, 4640, 4641, 8370, 8420, 8461, 8490, 8496, 8499; animals: 1440, 1441, 1460, 1463, 1500, 1510, 1540, 1590, 1600, 1603, 1604, 1610, 1710, 1721, 1722, 1731, 1740, 1750, 1811, 1920). Negative pictures (violent: 2683, 3500, 3530, 6212, 6250, 6312, 6313, 6315, 6350, 6360, 6530, 6540, 6550, 6560, 6571, 6821, 9423, 9424, 9427, 9428; negative: 2710, 2730, 2751, 3005.1, 3168, 3170, 3230, 3261, 3266, 6834, 8485, 9050, 9410, 9421, 9635.1, 9800, 9810, 9903, 9910, 9925; animals: 1019, 1050, 1052, 1111, 1201, 1205, 1220, 1274, 1275, 1525, 7359, 7380, 9140, 9180, 9181, 9182, 9560, 9561, 9570, 9571). Violent pictures depicted aggressive acts such as assaults with weapons and beatings, while nonviolent images depicted drug abuse, disease, and mutilations. Four pictures from each of the valence categories were used as sample pictures for practice with the task (IAPS numbers: 2331, 2575, 3030, 3301, 5836, 6243, 7550, 9210, 8350, 4649, 4650, 4669).]. Five of these included people, three included animals, and one included objects. The five picture types with people included neutral people, erotic heterosexual couples, positive (i.e., families and sporting events), violent (i.e., armed assaults, beatings), and negative (i.e., grief and loss, mutilations). The pictures of animals included neutral, positive, and negative stimuli. Four pictures from each of the valence categories were used as sample pictures for practice with the tasks.

**Table 1:** Mean Normative Arousal Ratings and 95% Confidence Intervals by Picture Type.

Picture Type	Subtype	*M*	95% CI
Neutral	Animal	5.15	±1.81
	Object	2.77	±1.20
	People	3.91	±0.99
Negative	Animal	5.64	±1.16
	Nonviolent	6.14	±1.21
	Violent	6.30	±1.01
Positive	Animal	4.38	±1.07
	Erotic	6.33	±0.55
	Families	5.46	±1.22

### Procedure

Participants came into the lab and the application of the Electro-cap was briefly explained, after which written informed consent was obtained. Participants then completed the Edinburgh Handedness Inventory [31]. After the Electro-cap was fitted to the participant, s/he was moved to the testing room and asked to sit comfortably in front of a computer monitor. Before data collection began the picture rating task was described, the participant was asked to limit head and eye movements during the task, and the experimenter answered any questions.

The task was presented on a computer using E-Prime 1.2 (Psychology Software Tools, Pittsburgh, PA). Participants responded with a keyboard and sat 100 cm from the monitor. Each of the 180 pictures was presented once in three different blocks, so the participants saw and rated each picture three times. In one block participants rated pictures for how pleasant they were, in another for how threatening they were, and in a third for how colorful (the amount of color present in the picture) they were on a scale from one (least) to four (most). The pictures remained on the screen until the participants made the rating and there was 1000 ms between the response and onset of the next picture. Pictures were 512×384 pixels at 1280×1024 pixel resolution on the monitor. The visual angle of the pictures was 7.85×5.73 degrees. Instructions were presented at the beginning of each block indicating how participants should rate the pictures. Individuals used the index and middle fingers of the left and right hands to respond. Participants were shown four of the sample pictures at the beginning of each block to practice rating. Order of the pictures within each block was random for every participant and the order of the three blocks was counterbalanced across participants.

### Electrophysiological Recording and Analysis

The electroencephalogram (EEG, filter.02–150 Hz, gain 1000, 16-bit A/D conversion) was recorded from an array of 68 tin electrodes sewn into an Electro-cap (Electro-Cap International, Eaton, OH) or affixed to the skin with an adhesive patch using a modified 10–10 system. The Electro-cap was interfaced to a DBPA-1 (Sensorium Inc., Charlotte VT) that amplified and digitized the data. Vertical and horizontal eye movements were recorded from electrodes placed next to and below the right and left eyes. During recording all electrodes were referenced to electrode Cz, then re-referenced to an average reference for data analysis. For the analyses of mean amplitude, a.1 to 20 Hz IIR bandpass filter was applied to the ERP data. Given the presence of considerable alpha activity in the later part of the analyzed epoch and the fact that affective valence did not have a consistent effect on the amplitude of ERP components preceding the EPN (i.e., N1 and P2), a.1 to 8 Hz IIR bandpass filter was applied to the data for the PLS analyses.

Ocular artifacts were corrected using a covariance-based technique including empirically derived estimates of the EEG associated with artifact and artifact free data (Source-Signal Imaging, San Diego). ERP analysis epochs were obtained offline and included -200 ms of prestimulus activity and 1200 ms of poststimulus activity. ERPs for each picture type were averaged for the rating tasks. Based upon previous research [3], [6]–[8], [18] or visual inspection of the ERP data, measurements of peak voltage were taken for 100 to 150 ms (N1) and 160 to 220 ms (P2); and measurements of mean voltage were taken for 200 to 250 ms (EPN) and 400 to 600 ms (LPP) post-stimulus onset. The measurement intervals represented the peak of the components in the grand-averaged data. Analyses of the N1 and P2 included data for electrodes O1, Oz, and O2; analyses of the EPN included data for electrodes PO9 and PO10; and analyses of the LPP included electrodes CPz and Pz.

### Partial Least Squares (PLS) Analysis

PLS analysis [28], [29] was applied to an ERP data matrix representing subjects, picture type and orienting task in the rows, and the amplitudes for time points between 0–1000 ms at 64 channels in the columns. The input matrix for the PLS analyses were obtained by mean-centering the columns of the data matrix with respect to the grand mean. Singular value decomposition (SVD) was performed on the deviation matrix to identify the structure of the latent variables. Three outputs were obtained from the SVD that were used to interpret the relationships between ERP amplitude and task design. The first was a vector of singular values – that are similar to eigenvalues and represent the unweighted magnitude of each latent variable – used to calculate the percentage of task-related variance attributable to each latent variable. The second and third outputs represent the structure of the latent variables and are orthogonal pairs of vectors (*saliences*) that are similar to component loadings. One vector defines the contrasts among conditions (*brain scores*) and the other vector represents the *ERP saliences* that reflect the temporal-spatial distribution of the latent variable across the scalp. The electrode saliences reflect components or modulations of the ERP waveforms that differ in amplitude across task conditions (e.g., a modulation of the LPP might reflect stable saliences over the central and parietal regions of the scalp between 400–1000 ms). The significance of the latent variables was determined using a permutation test (500 replications) that provided an exact probability of observing the singular value by chance; the stability of the ERP saliences at each time point and location on the scalp and the brain scores for the task conditions was established through bootstrap resampling (500 replications) that provided a standard error for each of the saliences or brain scores. The ratio of the salience to its bootstrapped standard error is approximately equivalent to a z-score; therefore, bootstrap ratios greater than 3.0 can be taken to indicate saliences that differ from zero at the *p*<.01 level.

## Results

Based upon the findings of Weinberg and Hajcak [1] the data for pictures of objects were excluded from the analyses. Also, given the results of this study demonstrating that the inclusion of people, and possibly faces, is critical when examining the neural correlates of affective picture processing [1]; the data for pictures including people and animals were considered in separate analyses. All inferential statistics were significant at the p<.05 level unless otherwise indicated.

### Behavioral Data

#### Pictures including people

The picture rating data were analyzed in a set of ANOVAs including data for neutral, negative, violent, positive, and erotic pictures (see Table 2 for mean ratings). Significant main effects of content were followed by paired post hoc contrasts representing ordinal changes in the means from lowest to highest ratings that were Bonferroni adjusted, *p*<.0125. This approach was adopted to minimize the number of post hoc comparisons required to quantify mean differences in the ratings. For the color rating task, the main effect was significant, *F*(4,156) = 30.77, *η_p_^2^* = .44. The colorfulness ratings of negative pictures did not differ from erotic pictures, *t*(39) = 1.59, nor did the ratings of erotic pictures differ from the violent pictures, *t*(39) <1. Neutral pictures received higher ratings of colorfulness than violent pictures, *t*(39) = 5.01, and positive pictures were rated as more colorful than neutral pictures, *t*(39) = 2.70. For the pleasantness rating task, the main effect was significant, *F*(4,156) = 148.79, *η_p_^2^* = .80. The ratings for negative and violent pictures were not significantly different, *t*(39) <1. Neutral pictures were rated as more pleasant than violent pictures, *t*(39) = 15.33. The ratings for neutral and erotic pictures did not differ, *t*(39) <1; the positive pictures were rated as more pleasant than the erotic pictures, *t*(39) = 4.27. For the threat rating task, the main effect was significant, *F*(4,156) = 226.38, *η_p_^2^* = .85. Threat ratings of positive and neutral pictures did not differ, *t*(39) = 2.21, nor did the ratings for neutral and erotic pictures differ, *t*(39) <1. Negative pictures were rated as more threatening than erotic pictures, *t*(39) = 13.43, and violent pictures were rated as more threatening than negative pictures, *t*(39) = 3.77.

**Table 2:** Mean Ratings of Each Picture Type by Orienting Task.

Picture Type	Subtype	Color (SE)	Threat (SE)	Pleasant (SE)
People	Neutral	2.96 (.36)	1.32 (.37)	2.81 (.48)
	Negative	2.25 (.56)	3.19 (.63)	1.23 (.36)
	Violent	2.44 (.64)	3.47 (.55)	1.25 (.29)
	Erotic	2.41 (.52)	1.39 (.57)	2.86 (.86)
	Positive	3.09 (.37)	1.22 (.29)	3.48 (.51)
Animals	Neutral	2.79 (.08)	2.37 (.10)	1.99 (.09)
	Negative	2.12 (.07)	2.72 (.11)	1.46 (.07)
	Positive	3.16 (.06)	1.21 (.05)	3.34 (.08)

#### Pictures including animals

For the color-rating task, the main effect was significant, *F*(2, 78) = 91.90, *η_p_^2^* = .70, and ratings increased from negative animals to neutral animals, *t*(39) = 10.09, and from neutral animals to positive animals, *t*(39) = 5.21. For the pleasantness rating task, the main effect was significant, *F*(2, 78) = 174.39, *η_p_^2^* = .82, and ratings increased from negative animals to neutral animals, *t*(39) = 7.59, and from neutral animals to positive animals, *t*(39) = −12.74. For the threat rating task, the main effect was significant, *F*(2, 78) = 155.81, *η_p_^2^* = .80, and ratings increased from positive animals to neutral animals, *t*(39) = 12.62, and from neutral to negative animals, *t*(39) = 5.93.

### Electrophysiological Data

The mean voltage data were analyzed in a set of 3 (rating task) × 3 or 5 (picture content) × 2 or 3 (electrode) ANOVAs. As in the analyses of the picture rating data, significant main effects of picture content were followed by paired post hoc contrasts representing ordinal changes in voltage from lowest to greatest amplitude that were Bonferroni adjusted, *p*<.0125.

#### Pictures including people

##### N1

**Figure 1:**
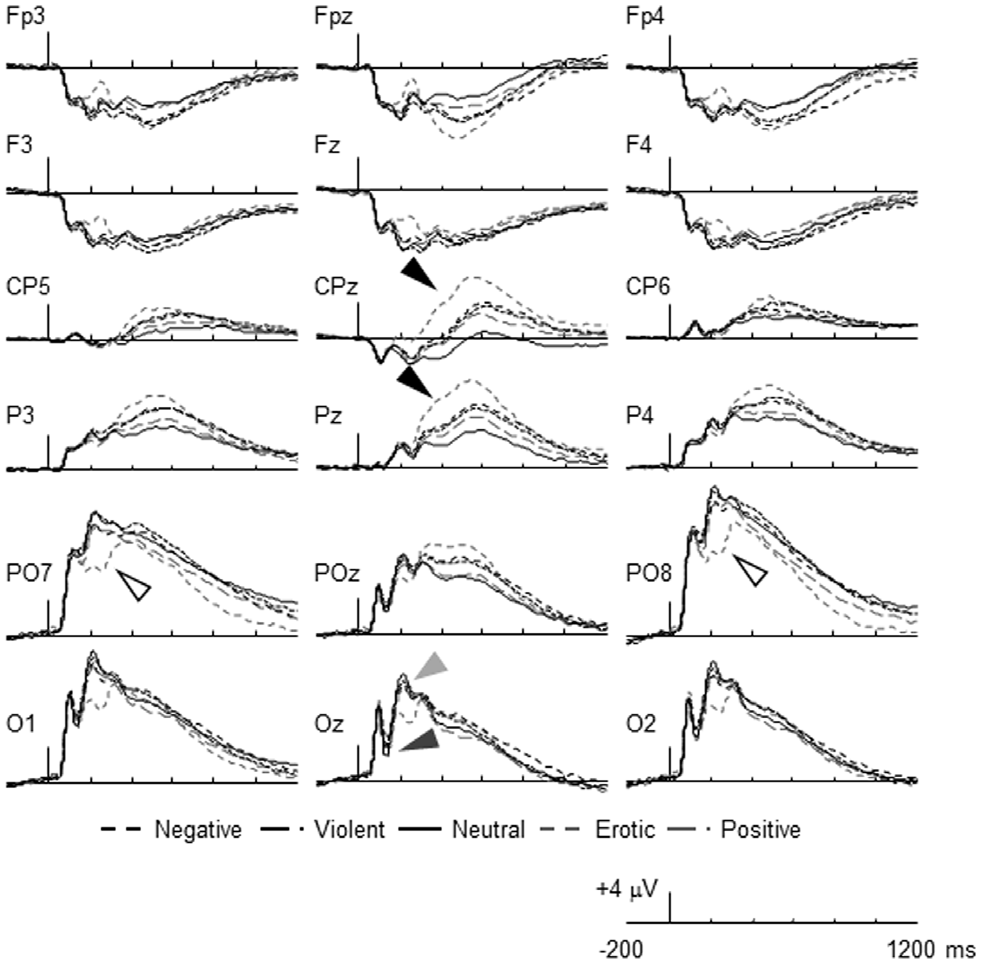
The grand-averaged ERPs collapsed across orienting tasks at select electrodes portraying the effect of picture content (i.e., affective valence) on the amplitude of the N1 (dark gray arrow), P2 (light gray arrow), EPN (open arrow), and LPP (filled arrow). The tall bar represents stimulus onset, the short bars represent 200 ms increments, and positive is plotted up.

The main effect of picture content was significant, *F*(4, 156) = 5.69, *η_p_^2^* = .13, *ε* = .67 (Figure 1 and Table 3). The amplitude of the N1 was marginally greater for positive than neutral pictures, *F*(1, 39) = 3.43, *η_p_^2^* = .08. None of the other contrasts were significant, *F*’s *<*1.25, *η_p_^2^*’s <.03. For the N1, the rating task x picture content interaction was not significant, *F* <1.00, *η_p_^2^* = .02. Given the pattern of mean differences for the five types of picture, it does not appear that there was a clear effect of valence on the N1.

**Table 3:** Mean Amplitude (µV) of the N1, P2, EPN, and LPP.

Picture Type	Subtype	N1 (SE)	P2 (SE)	EPN (SE)	LPP (SE)
People	Neutral	3.45 (4.86)	15.52 (6.63)	16.17 (1.06)	1.52 (.40)
	Negative	4.19 (4.68)	15.07 (6.65)	15.82 (1.07)	3.99 (.48)
	Violent	4.11 (4.35)	14.45 (6.38)	14.52 (.99)	4.48 (.49)
	Erotic	3.96 (4.67)	11.90 (6.47)	9.14 (1.03)	7.88 (.57)
	Positive	2.90 (4.59)	14.46 (6.82)	14.32 (1.04)	3.32 (.51)
Animals	Neutral	3.68 (4.89)	13.80 (6.37)	14.03 (1.11)	4.55 (.48)
	Negative	3.84 (4.59)	14.24 (6.47)	14.99 (1.12)	4.17 (.44)
	Positive	3.94 (4.49)	15.18 (6.60)	14.74 (1.14)	3.76 (.39)

#### P2

The main effect of picture content was significant, *F*(4, 156) = 26.49, *η_p_^2^* = .40, *ε* = .64 (Figure 1 and Table 3). The amplitude of the P2 differed between erotic and positive pictures, *F*(1, 39) = 42.74, *η_p_^2^* = .52, did not differ between positive and violent pictures, *F* <1.00, *η_p_^2^* = .001, differed between violent and negative pictures, *F*(1, 39) = 4.96, *η_p_^2^* = .11, and did not differ between negative and neutral pictures, *F*(1, 39) = 2.30, *η_p_^2^* = .06. The rating task x picture content interaction was not significant, *F* <1.00, *η_p_^2^* = .01. The results of these analyses reveal that valence was associated with a reduction in the amplitude of the P2, and that this was particularly true for erotic pictures.

#### EPN

The main effect of picture content was significant, *F*(4, 156) = 82.14, *η_p_^2^* = .55, *ε* = .68 (Figure 1 and Table 3). The amplitude of the EPN did not differ between neutral and negative pictures, *F*(1, 39) = 1.60, *η_p_^2^* = .039, differed between negative and violent pictures, *F*(1, 39) = 21.64, *η_p_^2^* = .36, did not differ between violent and positive pictures, *F* <1.00, *η_p_^2^* = .012, and differed between positive pictures and erotic pictures, *F*(1, 39) = 103.73, *η_p_^2^* = .73. The rating task x picture content interaction was not significant, *F*(8, 312) = 1.46, *η_p_^2^* = .001. The results of this analysis reveal a general effect of valence on the EPN that was much stronger for erotic pictures that the other stimuli.

#### LPP

The main effect of picture content was significant, *F*(4, 156) = 114.89, *η_p_^2^* = .75, *ε* = .90 (Figure 1 and Table 3). The amplitude of the LPP differed between neutral and positive pictures, *F*(1, 39) = 36.69, *η_p_^2^* = .49, did not differ between positive and negative pictures, *F*(1, 39) = 4.24, *η_p_^2^* = .10, or negative and violent pictures, *F*(1, 39) = 3.95, *η_p_^2^* = .09, and differed between violent and erotic pictures, *F*(1, 39) = 150.75, *η_p_^2^* = .79. The rating task x picture content interaction was also significant, *F*(8, 312) = 2.16, *η_p_^2^* = .05, *ε* = 1.0. Post hoc analyses examining the effect of rating task on each of the pictures types revealed that the amplitude of the LPP for erotic pictures was sensitive to task, *F*(2, 78) = 4.12, *η_p_^2^* = .10, *p* = .02, being greater in the pleasantness-rating task than the colorfulness-, *F*(1, 39) = 4.80, *η_p_^2^* = .11, or threatening-rating tasks, *F*(1, 39) = 6.22, *η_p_^2^* = .14, and not differing in the latter two tasks, *F* <1.00, *η_p_^2^* = .023. The effect of rating task was not significant for the other picture types, *F’s*(2, 78) <2.23, *p’s* >.11, *η_p_^2^*<.054.

In order to make a more direct comparison between the effect of valence on the LPP in the current study and past research [5], [12], [16], [18], the data for erotic pictures and positive pictures were collapsed and compared to the combined negative pictures (i.e., violent and negative) and neutral pictures. The analysis represented a 3 (rating task) × 3 (picture content: positive, negative, neutral) × 2 (electrode: CPz, Pz) ANOVA. The main effect of picture content was significant, *F*(2, 76) = 125.18, *η_p_^2^* = .76, *ε* = .92. The amplitude of the LPP was greater for positive pictures (*M* = 5.60 µV, *SE* = .51) than for negative pictures (*M* = 4.32 µV, *SE* = .47), *F*(1, 39) = 36.45, *η_p_^2^* = .48, and was greater for negative pictures than for neutral pictures (*M* = 1.52 µV, *SE* = .40), *F*(1, 39) = 118.17, *η_p_^2^* = .75. These findings converge with previous research demonstrating that the negativity bias may not be observed when analyzing mean voltage with erotic and other positive pictures combined [1], [2].

#### Pictures including animals

**Figure 2:**
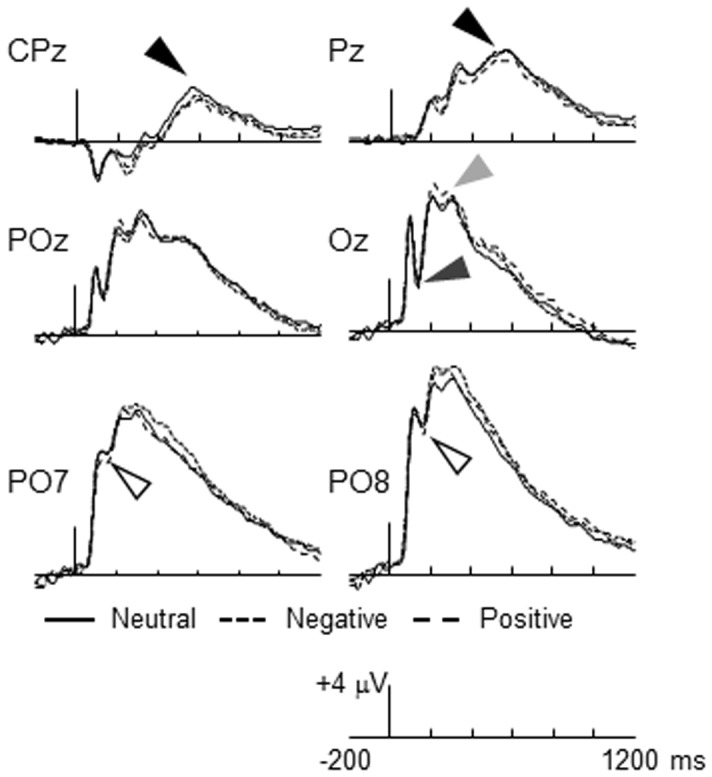
The grand-averaged ERPs at select electrodes portraying the amplitude of the N1 (dark gray arrow), P2 (light gray arrow), EPN (open arrow), and LPP (filled arrow) for animal pictures in the color-rating task. The tall bar represents stimulus onset, the short bars represent 200 ms increments, and positive is plotted up.

The grand-averaged ERPs for the pictures of neutral, positive and negative animals collapsed across rating tasks are presented in Figure 2.

#### N1

The valence of the animal pictures did not appear to influence the amplitude of the N1. The effect of picture content was not significant, *F* <1.0, *η_p_^2^* = .02, *ε* = 1.0. The effect of task and the task x content interaction were also not significant, *F*’s <2.86, *η_p_^2^*’s <.06.

#### P2

The amplitude of the P2 appeared to be greater for positive animal pictures. The effect of picture content was significant, *F*(2, 78) = 10.25, *η_p_^2^* = .21, *ε* = .95. Further analysis revealed that the amplitude of the P2 did not differ significantly for negative and neutral animals, *F*(1, 39) = 2.87, *η_p_^2^* = .07. The amplitude of the P2 was greater for positive animals than for neutral animals, *F*(1, 39) = 16.49, *η_p_^2^* = .30. The effect of task and the task x content interaction were not significant, *F*’s <1.0, *η_p_^2^*’s <.01.

#### EPN

The amplitude of the EPN did not appear to differentiate pictures of negative, positive, and neutral animals. The effect of picture content was not significant, *F* <1.0, *η_p_^2^* = .02, *ε* = 1.0. The effect of task and the task by content interaction were also non-significant, *F’s* <1.0, *η_p_^2^*’s <.02.

#### LPP

The amplitude of the LPP did not appear to be sensitive to the valence of the animal pictures. The effect of picture content was not significant, *F* <1.0, *η_p_^2^* = .02, *ε* = 1.0. The effect of task and the task by content interaction were also non-significant, *F’s* <2.2, *η_p_^2^*’s <.06.

### PLS Analysis

The results described in the previous section converge with the findings of Briggs and Martin [2] and Weinberg and Hajcak [1] in revealing that the amplitude of both the EPN and LPP were greatest for erotic pictures and that the presence or absence of the negativity bias was dependent upon whether negative pictures were compared to positive pictures or erotic pictures. Based upon these data one might conclude that there are not distinct neural correlates of processing negative and positive stimuli during affective picture processing. However, one potential limitation of the analyses of mean voltage is that this method does not take into account the spatial-temporal overlap of components of the ERPs that may be differentially sensitive to various aspects of affective information processing [8]. Thus, it is possible that different patterns of neural recruitment associated with processing negative and positive pictures were obscured in the analyses of mean voltage. To examine this possibility a PLS analysis was conducted that included data for the neutral, negative, violent, positive, and erotic pictures including people in the three orienting tasks to determine whether the we could distinguish the neural correlates of processing positive and negative pictures. Given the general absence of mean differences in amplitude for the animal pictures, these data were not examined further using PLS analysis.

**Figure 3:**
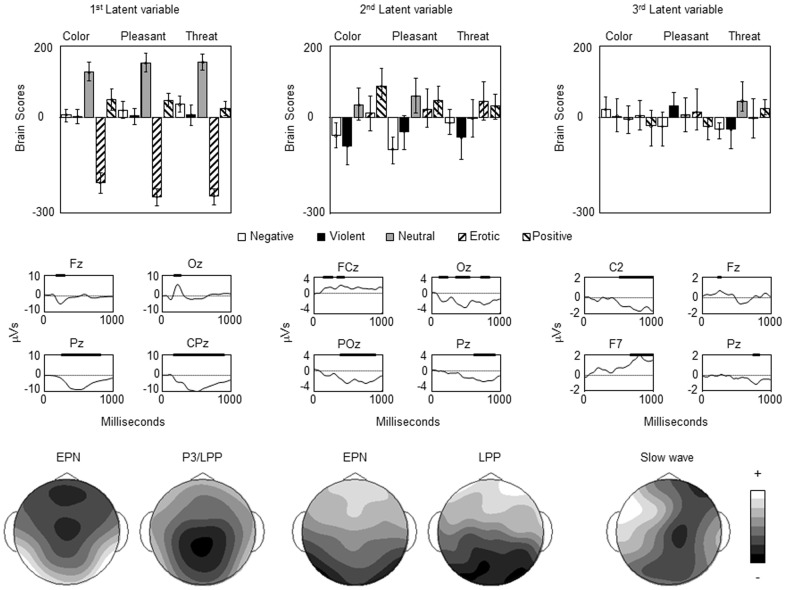
Results of the PLS analysis. The upper panel portrays the contrasts (brain scores) for the first three latent variables representing significant mean differences in ERP amplitude across picture type and orienting task. The errors represent the 95% confidence intervals from the bootstrap test. The middle panel portrays the electrode saliences at four select electrodes for each latent variable. Note that the polarity of the EPN and P3 is reversed from the scalp data given the negative brain score for erotic pictures. The (o) above the x-axis indicate electrode saliences where the bootstrap ratio exceeded 7.0, 4.0 or 2.5 for the first to third latent variables, respectively. Differences in the threshold for the bootstrap test across the three latent variables were used given the dramatic difference in the strength of the latent variables. The lower panel portrays scalp topography maps designed to highlight the distribution of the primary components of the ERPs that were expressed in the three latent variables. For the first latent variable the maps represent ERP activity at 250 ms (EPN) and 500 ms (P3) after stimulus onset; for the second latent variable the maps represent the ERP activity at 200 ms (EPN) and 760 ms (LPP); and for the third latent variable the maps represent ERP activity at 800 ms (slow wave). Lighter colors reflect positive voltage and darker colors reflect negative voltage. The scale varies across maps given differences in the strength of the three latent variables.

The PLS analysis revealed two significant latent variables (*p*’s <.01) and a third that was marginally significant (*p* = .06) that accounted for 72.03%, 14.38%, and 3.09% of the cross-block covariance, respectively. The first latent variable reflected a contrast between erotic pictures and neutral and positive pictures across the three tasks (Figure 3). The electrode saliences revealed two stable periods of neural activity for this latent variable. The first represented a transient modulation of the ERPs over the posterior region, likely reflecting an effect on the EPN, and the frontal region (e.g., electrode Fz in Figures 1 and 3) that peaked around 250 ms after stimulus onset. The second represented a sustained modulation of the ERPs extending from the central-parietal to the parietal region between 200–800 ms after stimulus onset, likely reflecting an effect on the P3. The contrast was stronger for the pleasantness- and threat-rating tasks indicating that this latent variable was sensitive to the relevance of the affective information contained in the stimuli. Given the expression of the EPN and P3 components in the first latent variable, this effect may reflect variation in the allocation of attention to erotic pictures that is enhanced when emotion is relevant to task performance.

The second and third latent variables appeared to capture the neural correlates processing negative pictures across the three tasks (Figure 3). The nature of the contrast for the second latent variable differed across the three orienting tasks, reflecting differences between negative and violent pictures and positive pictures for the color-rating task, differences between negative pictures and positive and neutral pictures in the pleasantness-rating task, and violent pictures in the threat-rating tasks. The electrode saliences revealed stable neural activity over the posterior and frontal regions between 200–300 ms after stimulus onset that likely reflects an effect on the EPN, and more sustained neural activity between 400–900 ms after stimulus onset extending from the occipital to the parietal regions reflecting the LPP [10]. Given the pattern of brain scores and electrode saliences for the second latent variable, this effect may reflect the early allocation of attention to negative pictures coupled with more sustained evaluative processing of these stimuli that varies with the information processing demands of the task.

The third latent variable reflected a contrast between negative and violent pictures with neutral and positive picture in the threat-rating tasks. The electrode saliences for this latent variable revealed a small transient modulation of the ERPs over the frontal region around 200 ms after stimulus onset, and a sustained modulation of the ERPs over the left lateral frontal and right central regions between roughly 600–1000 ms after stimulus onset. The third latent variable accounted for relatively little variance and was only marginally significant, indicating that this effect should be replicated in future research.

## Discussion

The current study was designed to determine whether or not there are neural correlates of affective picture processing that distinguish the processing of negative pictures from that of positive pictures while considering the influence of sexually arousing stimuli and other types of positive pictures. When people were portrayed in the pictures, the analyses of mean voltage revealed that the amplitude of the N1 was greater for neutral and positive pictures than for erotic, violent, and negative pictures. This finding differs from previous research, and could be related to the content of the neutral pictures (i.e., the current study used pictures of people, previous studies used pictures of objects). These analyses also revealed effects of valence on the EPN and the LPP that were clearly related to differences in picture content, and were particularly sensitive to erotic pictures. The PLS analysis revealed one latent variable associated with the neural correlates of processing erotic pictures, and two latent variables associated with the neural correlates of processing negative pictures. These findings are consistent with other recent evidence revealing differences in the ERP correlates of processing negative and positive pictures [10]. In contrast to pictures including people, we did not observe differences in the amplitude of the ERPs for pictures of animals that varied in valence, with the exception that the amplitude of the P2 was marginally greater for positive pictures than for neutral or negative pictures. This finding is consistent with the idea that the presence of people, and possibly human faces, is important for observing effects of valence during affective picture processing [1].

While the negativity bias has been proposed to be a universal characteristic of affective information processing [9], [21], [23], data reported by Briggs and Martin [2] and Weinberg and Hajcak [1] question this assumption. Here we replicated the findings of these two recent studies, demonstrating that the amplitude of the EPN and LPP were greatest for erotic pictures. Additionally, when the data for positive and erotic pictures were collapsed and compared to the negative pictures, a positivity bias was observed (i.e., the amplitude of the LPP was greater for positive pictures than for negative pictures). Based on current and other recent findings, it appears that depending upon the distribution of sexually arousing and other types of positive pictures selected for a given study one could observe a negativity bias [9], a positivity bias ([16] and current data), or no difference between positive and negative pictures in the scalp recorded data. Furthermore, the results of the analyses of *mean differences in amplitude* are consistent with the idea that the negativity bias may reflect a stimulus artifact rather than representing a distinct component of affective information processing [1], [2].

In contrast to the results of the analyses of mean voltage, the results of the PLS analysis appear to support a different set of conclusions. In this analysis, the first latent variable contrasted the ERPs elicited by erotic pictures with those elicited by positive and neutral pictures in each of the three orienting tasks. The second and third latent variables contrasted the ERPs elicited by negative and violent pictures with those elicited by positive pictures and to a lesser degree neutral pictures, with the third latent variable reflecting the processing of negative pictures in the threat-rating task. These findings lead to the suggestion that processing erotic and negative pictures is associated with different patterns of neural recruitment, and that the neural correlates of processing negative pictures are sensitive to picture content and the processing demands of the orienting task [10].

An examination of the distribution of the electrode saliences for the three latent variables leads to the suggestion that both overlapping and different components of the ERPs are associated with the processing of erotic and negative pictures. The first and second latent variables revealed stable neural activity over the posterior and frontal regions of the scalp around 200–300 ms after stimulus onset that likely reflects an effect on the EPN and an associated frontal positivity. This finding leads to the suggestion that the EPN reflects a core element of affective picture processing that is common to both positive and negative valence [8].

In contrast to the EPN, there were differences in the time course and topography of the ERPs associated with processing erotic pictures and negative pictures beginning around 300 ms after stimulus onset. The first latent variable revealed stable electrode saliences between 200–800 ms after stimulus onset over the central-parietal and parietal regions of the scalp that may reflect an effect on the P3. The distribution of the stable electrode saliences is consistent with the data reported by Briggs and Martin ([2] Figure 1) where the difference between erotic and negative pictures appears to be greatest around electrode CPz. The stable electrode saliences for the second latent variable in the later part of the epoch differed from those of the first latent variable in a number of ways. Over the posterior region the stable electrode saliences for this latent variable were stronger over the parietal-occipital region than the central-parietal region and this effect was later for the second than the first latent variable. The stable electrode saliences for the third latent variable reflected a sustained modulation of the ERPs over the left lateral frontal and right central regions of the scalp that differed in distribution from both the P3 and the LPP associated with the first and second latent variables, respectively. Together, these findings lead to the conclusion that different neural generators distributed across central and posterior cortical structures may be involved in the processing of positive and negative valence [10], [32]–[34].

The effects of picture content on the EPN and LPP were generally similar across the three orienting tasks in the analyses of mean voltage, with the exception that the amplitude of the LPP for erotic pictures was slightly greater for the pleasantness-rating task when all participants were included in the analyses. The pattern of brain scores for the first latent variable converges with this finding, as the contrast was stronger in the pleasantness- and threat-rating tasks than in the color-rating task. These finding converge with previous work revealing that directing attention to affective versus non-affective aspects of a stimulus can modulate the amplitude of the LPP [17], [19], [20]. Furthermore, differences in the effect of rating task on processing the erotic pictures and the other valenced pictures may indicate that appraisal has somewhat different effects on the neural correlates of affective picture processing depending upon picture content.

The neural correlates of processing negative pictures varied with picture content and orienting tasks in the PLS analysis. For the second latent variable, the effect was stable for both negative and violent pictures in the color-rating task, was limited to negative pictures in the pleasantness-rating task, and was limited to the violent pictures for the threat-rating task. For the third latent variable the differences between negative and neutral/positive pictures were limited to the threat-rating task. These findings converge with other recent evidence from our laboratory [10], and demonstrate that the ERP correlates of processing negative pictures are sensitive to both stimulus-driven and top-down attentional or decision processes, rather than reflecting relatively automatic processing [9], [21], [23].

The results of the mean voltage and PLS analyses provide some insight into the nature of the processes associated with the EPN and LPP. The mean voltage analysis revealed that the amplitude of the EPN was greater for erotic pictures than for negative or violent pictures. This finding indicates that the EPN must be sensitive to processes other than arousal [5], [12] since the erotic, negative and violent pictures were well matched for normative levels of arousal. The amplitude of the EPN was not affected by rating task in the analyses of mean voltage; this finding may indicate that the processes contributing to the EPN are not particularly sensitive to “top-down” attentional or decision processes that influenced the amplitude of the LPP. The EPN was expressed in the first and second latent variables that were differentially sensitive to the processing of erotic and negative stimuli. Together these findings lead to the suggestion that the EPN is associated with processes that are related to stimulus-driven orienting towards positive and negative stimuli that may be relevant to basic motivational processes associated with survival [5], [12], [13].

In contrast to the EPN, the LPP reflected multiple components of the ERPs that were differentially sensitive to erotic and negative pictures, and that were modulated by the orienting task. This finding converges with evidence from a study using PCA revealing that multiple components of the ERPs contributed to the LPP [8]. Also, the various components that contribute to the LPP were sensitive to the influence of attentional or decision processes that varied across the three rating tasks [17]. Together, these findings dissociate the neuro-cognitive processes associated with the EPN and LPP and lead to the suggestion that the EPN reflects stimulus-driven processes associated with orienting to motivationally relevant stimuli while the LPP reflects goal-directed attentional or decision processes contributing to affective information processing.

There are some limitations of the study that must be considered. First, the number of trials contributing to the ERPs for each picture type is relatively small (i.e., 20). This could make it difficult to observe robust effects of picture valence on early components of the ERPs (e.g., N1 and P2). In anticipation of this limitation of the data we included a relatively large sample in the study. Also, it is worth noting that our primary interest in the study was related to the effects of picture type and orienting task on the EPN and LPP so we fully acknowledge that the design may not be optimal for examining components of the ERPs elicited during the first couple hundred milliseconds of stimulus processing. Second, similar to other statistical techniques using singular value decomposition (e.g., PCA; [8]), PLS analysis could be limited in the ability to identify topographic differences in neural activity that are sensitive to variation in stimulus valence. One issue is related to whether the latent variables capture differences in the topography of the neural correlates of affective picture processing; or instead express latency differences in components across conditions [35]. At a general level, Lobaugh et al. [28] demonstrated that PLS can identify systematic variation in both the latency and distribution of experimental effects across task conditions. In the context of the current study, Figure 2 reveals clear differences in the topography of the stable electrode saliences for the later neural activity extending from the central to the parietal-occipital regions that distinguished the first and second latent variables. Additionally, other recent data from our laboratory [10] revealed two to three significant latent variables that were differentially sensitive to the negativity bias and processing positive pictures, and revealed differences in topography over the posterior region of the scalp. Together, the data from these two studies lead us to believe that PLS analysis provides a robust method for identifying the neural correlates of aspects of affective picture processing that are differentially sensitive to stimulus valence.

In summary, the findings from the current study lead to the suggestion that processing sexually arousing and negative pictures are associated with different patterns of neural activity. Consistent with recent work [10], [19], [20], the demands of the orienting task influenced the amplitude of the neural correlates of the processing erotic stimuli, and the time course, topography, and functional characteristics (i.e., pattern of brain scores) of the neural correlates of processing negative pictures. These findings lead to the suggestion that the neural correlates of affective picture processing are sensitive to both relatively automatic, and attentional or decision processes.
